# Survival benefit of hepatic resection versus transarterial chemoembolization for hepatocellular carcinoma with portal vein tumor thrombus: a systematic review and meta-analysis

**DOI:** 10.1186/s12885-017-3895-z

**Published:** 2017-12-28

**Authors:** Xiu-Ping Zhang, Kang Wang, Nan Li, Cheng-Qian Zhong, Xu-Biao Wei, Yu-Qiang Cheng, Yu-Zhen Gao, Han Wang, Shu-Qun Cheng

**Affiliations:** 1 Department of Hepatic Surgery VI, Eastern Hepatobiliary Surgery Hospital, Second Military Medical University, 225 Changhai Road, Shanghai, 200433 China; 2 Department of Laboratory Medicine, Eastern Hepatobiliary Surgery Hospital, Second Military Medical University, Shanghai, China; 3 Department of Pathology, Eastern Hepatobiliary Surgery Hospital, Second Military Medical University, Shanghai, China

**Keywords:** Hepatic resection, Transarterial chemoembolization, Hepatocellular carcinoma, Portal vein tumor thrombus

## Abstract

**Background:**

No consensus treatment has been reached for hepatocellular carcinoma (HCC) with portal vein tumor thrombus (PVTT). Hepatic resection (HR) and transarterial chemoembolization (TACE) have been recommended as effective options, but which is better remains unclear. This meta-analysis is to compare the effectiveness of HR and TACE for HCC with PVTT patients.

**Methods:**

The PubMed, EMBASE, Cochrane Library, VIP, Wan Fang, and Sino Med databases were systematically searched for comparing HR and TACE treating PVTT.

**Results:**

Twelve retrospective studies with 3129 patients were included. A meta-analysis of 11 studies suggested that the 1-, 2-, 3-, and 5-year overall survival (OS) rates (OR = 0.48, 95% CI = 0.41–0.57, I^2^ = 37%, *P* < 0.00001; OR = 0.21, 95% CI = 0.12–0.38, I^2^ = 43%, *P* < 0.00001; OR = 0.35, 95% CI = 0.28–0.44, I^2^ = 53%, *P* < 0.00001; OR = 0.28, 95% CI = 0.14–0.54, I^2^ = 72%, *P* = 0.0001, respectively) favored HR over TACE. In a subgroup analysis, HR had better 1-, 2-,3, 5-year OS for type I PVTT (OR = 0.33, 95% CI = 0.17–0.64, I^2^ = 20%, *P* = 0.001; OR = 0.32, 95% CI = 0.16–0.63, I2 = 0%, *P* = 0.001; OR = 0.18, 95% CI = 0.09–0.36, I2 = 0%, *P* < 0.00001; OR = 0.07, 95% CI = 0.01–0.32, I2 = 0%, *P* = 0.0006, respectively) and better 1-, 3-, and 5-year OS for type II PVTT (OR = 0.37, 95% CI = 0.20–0.70, I^2^ = 59%, *P* = 0.002; OR = 0.22, 95% CI = 0.13–0.39, I^2^ = 0%, *P* < 0.00001; OR = 0.16; 95% CI = 0.03–0.91; I^2^ = 51%, *P* = 0.04, respectively). There was no difference in 1-, 3-, or 5-year OS between HR and TACE for type III PVTT (OR = 0.86, 95% CI = 0.61–1.21, I^2^ = 0%, *P* = 0.39; OR = 0.83, 95% CI = 0.42–1.64, I^2^ = 0%, *P* = 0.59; OR = 0.59, 95% CI = 0.06–-6.04, I^2^ = 65%, *P* = 0.66, respectively).

**Conclusions:**

HR may lead to longer OS for some selected HCC patients with PVTT than TACE, especially for type I or II PVTT, with less difference being observed for type III or IV PVTT.

## Background

Hepatocellular carcinoma (HCC) is one of the most common types of cancer and has dismal outcomes with high morbidity and mortality [1]. Portal vein tumor thrombosis (PVTT) is a commonly recognized independent risk factor for HCC prognosis, occurring in 44–62.2% of these patients and being associated with a natural median survival time (MST) of 2.7–4 months [2] without any treatment interventions. According to Barcelona Clinic Liver Cancer (BCLC) guidelines [3], sorafenib is the only recommended treatment for PVTT, and the reported median survival time (MST) of patients treated with sorafenib is as short as 10.7 months [4]. However, multimodal treatments, such as hepatic resection (HR) and transarterial chemoembolization (TACE), have been widely applied to PVTT and have shown a survival benefit in patients with HCC in Asian countries [5–7]. At present, treatment strategies for HCC patients with PVTT remain controversial.

Due to recent advances in perioperative management and surgical techniques, HR has become a reasonably safe treatment option. Aggressive HR for HCC with PVTT has been proposed by several tertiary centers [6, 8, 9]. Similarly, TACE provides favorable long-term survival outcomes in advanced HCC patients with PVTT compared with the best supportive treatments if they have adequate liver function [7, 10]. However, the number of patients enrolled in these studies has generally been small, and the reports suffer from substantial selection bias. Therefore, whether to select HR or TACE as an initial treatment for these patients remains unclear [11–13]. Unfortunately, there is no reported systematic review or meta-analysis on the above controversy.

Here, we present the first systematic review and meta-analysis comparing HR and TACE for HCC with PVTT, with a focus on different types of PVTT.

## Methods

### Search strategy

Following the Preferred Reporting Items for Systematic Reviews and Meta-Analyses (PRISMA) guidelines [14], we systematically searched the PubMed, Cochrane Library, EMBASE, Web of Science, Chinese National Knowledge Infrastructure (CNKI), VIP, Wan Fang, and Sino Med databases with no limitations on language. Meanwhile, we comprehensively searched ClinicalTrials.gov to attain available outcomes of ongoing studies comparing HR with TACE for PVTT. The search was updated on January 1, 2017. The following search terms were used: “liver surgery” or “hepatic resection” or “surgical resection” AND “transcatheter arterial chemoembolization” or “TA(C)E” or “transarterial chemoembolization” or “chemoemboli*” or “emboli*” AND “(liver or hepatic or hepatocellular or hepatocellular) and (carcinom* OR cancer OR neoplasm* OR malign* OR tumor* OR tumour*)” or “HCC” or “hepatoma*” AND “portal vein tumor thrombus” or “(portal vein thrombosis)” or “PVTT”. All abstracts were independently screened by Zhang XP and Wang K, and full-text reports of the included papers were obtained for another screen.

### Study selection

#### Inclusion criteria

This meta-analysis was focused on comparing the efficacy and safety of HR versus TACE in the treatment of HCC patients with PVTT. Therefore, only comparative analysis concerning clinical value of HR alone versus TACE alone for HCC patients with PVTT was used. The inclusion criteria should be: (1) HCC patients with various types of PVTT who underwent HR or TACE without other treatments. (2) Clinical trials comparing the therapeutic effect of HR with TACE for these patients. (3) Trials including original data, such as 6-month or 1,2,3,5-years’ overall survival (OS), (DFS) and odds ratios (OR) or hazard ratio estimates (HRs) with 95% confidence intervals (95% CIs). (3) Relevant degree papers, conference summaries and abstracts, and some ongoing randomized controlled trials (RCTs) about HR or TACE for PVTT, with no publication language limitation applied.

#### Exclusion criteria

The exclusion criteria should be: (1) Advanced HCC patients without PVTT. (2) These patients receiving other treatments or combined treatments instead of HR or TACE alone. (3) Narrative reviews, case reports, current affairs review, letters, comments, or studies unrelated to our topics. (4) Repeated papers or papers that did not provide the necessary information.

### Data extraction and quality assessment

Two authors (Zhang XP and Wang K) of this article independently extracted and checked all data from the included papers. If necessary, a third author (Li N) was invited to participate in resolving disagreements through discussion and consensus. The following data were extracted:

1. Basic data from the article, including country, study design, authors, patient characteristics, methods and procedures of TACE or HR.
2. Basic data from patients with HCC, including therapy outcomes for HCC with PVTT, and the outcomes of patients undergoing HR or TACE for various PVTT types.

Some data were calculated, such as study methods and OS outcomes in different years, recurrence rate and DFS, some measures related to different PVTT subgroups, and OR estimates with 95% CIs.

Three authors of this article together extracted the data with a consensus and then entered the requisite data into RevMan software, version 5.3 (The Cochrane Collaboration, http://tech.cochrane.org). For nonrandomized controlled trials (NRCTs), the quality of the studies included in the meta-analysis was assessed using the Newcastle-Ottawa Scale (NOS) (The Ottawa Hospital: Research Institute. 2009. Available from URL: http://www.ohri.ca/programs/clinical_epidemiology/oxford.asp). In the NOS, if the quality score of an article is greater than or equal to 6 with a full score of 9, then the article is considered to be high quality. Publication bias was assessed with funnel plots, Begg’s test and Egger’s test [15], with a *P*-value <0.05 judged as statistically significant. All meta-analyses had good reliability and were not influenced by any one of the included studies based on calculations using RevMan software, version 5.3.

### Statistical analysis

The outcomes included OS rate, DFS, and outcomes of different types of PVTT. The included data are presented as OR estimates with 95% CIs for all outcomes. OS rates were assessed for different years, with some data being obtained from survival curves. The RRs of each study were pooled using a fixed effects model or a random effects model with RevMan version 5.3.

According to the suggestions of the Cochrane collaboration, Q statistics and the I2 index were used to assess heterogeneity, with significant heterogeneity indicated at *P* < 0.05 and an I2-index >50% [16]. The estimates were pooled with a fixed effects model if no significant heterogeneity was identified, whereas a random effects model was used for estimates with heterogeneity. Subgroup analyses were performed according to PVTT type.

## Results

### Identification of eligible studies

**Fig. 1:**
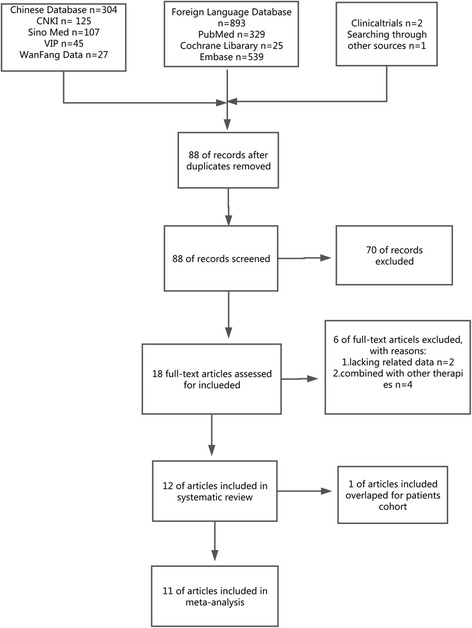
PRISMA flow diagram of the process used to identify eligible studies. CNKI: Chinese National Knowledge Infrastructure; VIP: Chongqing VIP Database for Chinese Technical Periodicals; Wan Fang: Wan Fang Database; Sino Med: Chinese Biological Medical Literature Database

Using our search strategy, we identified 1200 relevant studies, of which 1112 duplicates were excluded. Another 70 articles were excluded after the titles and abstracts were reviewed. Six studies were excluded for not meeting the requirements, such as the use of additional therapies and a lack of basic data, as shown in Fig. 1. At last, 12 retrospective controlled studies [11–13, 17–25] meeting the inclusion standards and involving 3129 patients were eligible for inclusion in the systematic review. The meta-analysis assessed 11 of these articles because one article had an overlapping patient cohort from 1997 to 2000.

### Patient characteristics

Table 1 presents the baseline characteristics of the patients in the included studies. The 12 studies were published from 2001 to 2016. A total of 3129 HCC patients with PVTT were included, of whom 1483 received HR and 1646 received TACE as an initial treatment. More men than women with HCC and PVTT were included in the analysis. Tumor size mostly ranged from 5 to 10 cm. Most tumors were single. Type I and II PVTT were most common and were determined using Cheng’s Classification [26, 27]. The baseline liver function for most participants was Child-Pugh A or B. Ten studies reported mostly HBV virology for HCC patients [11–13, 17–19, 22–25]. Serum AFP, a diagnostic marker of HCC, was more than 400 mg/L in 10 studies [11–13, 17–19, 22–25]. Specific details of the patients’ characteristics are recorded in Table 1.

**Table 1:** Basic characteristics of patients

Study(year)	Study design	Treatment	Country	Patients	AGE	Sex(M/F)	Tumor size(cm) (<5/≥5)	Tumor Number (S/M)	^a^Type of PVTT (I/II/III/IV)	Child-Pugh (A/B/C)	Virology HBV/Other	Cirrhosis (Yes/No)	Total bilirubin level (μmol/L)	Serum albumin level (g/L)	AFP(mg/L) (<400/≥400)
Fan 2001 [20]	R(1996-1998)	HR	China	74	48.2(20-70)	NA	35/39 (**≥**10/<10)	46/28	0/58/16/0	NA	NA	NA	NA	NA	NA
		TACE		18	48.2(20-70)	NA	13/5(**≥**10/<10)	13/5	0/12/6/0	NA	NA	NA	NA	NA	NA
Fan 2003 [19]	R(1997-2000)	HR	China	19	47 (26-75) (All)^b^	122/16 (All)	9/10 (**≥**10/<10)	10/9	0/14/5/0	105/33/0 (All)	103/35 (All)	NA	NA	NA	108/30 (**≥**10/<10) ng/ml(All)
		TACE		41	47 (26-75) (All)	122/16 (All)	21/20 (**≥**10/<10)	26/15	0/26/15/0	105/33/0 (All)	103/35 (All)	NA	NA	NA	108/30 (**≥**10/<10) ng/ml(All)
Cheng 2005 [18]	R(2000-2003)	HR	China	7	47.5 ± 6.9	5/2	62.7 ± 49.8 (mm)	7/0	2/4/1/0	NA	6/1/	NA	NA	NA	5/2(**≥**20/<20) μg/L
		TACE		38	47.9 ± 10.6	35/3	62.7 ± 49.8 (mm)	30/8	6/11/20/1	NA	32/6	NA	NA	NA	28/10 (**≥**20/<20)μg/L
Fan 2005 [21]	R(1997-2002)	HR	China	24	15/9 (**≥**45/<45)	20/4	11/13 (**≥**10/<10)	14/10	0/16/8/0	18/6/0	NA	NA	NA	NA	NA
		TACE		53	25/28 (**≥**45/<45)	49/4	28/25 (**≥**10/<10)	33/20	0/30/23/0	39/14/0	NA	NA	NA	NA	NA
Zhou 2011 [25]	R(2003-2010)	HR	China	38	22/16 (**≥**50/<50)	35/3	13/25	27/11	0/25/13/0	27/11/0	36/2	14/24	NA	NA	14/24
		TACE		10	5/5 (**≥**50/<50)	10/0	8/2	3/7	0/4/6/0	4/6/0	10/0	9/1	NA	NA	1/9
Peng 2012 [12]	R(2002-2007)	HR	HongKong China	201	55 (25-75)	187/14	76/125	95/106	27/68/83/23	197/4/0	172/27	176/25	12.9	36.8	562.3
		TACE		402	55 (23-75)	374/28	178/224	132/270	54/136/166/46	389/13/0	356/46	363/39^c^	12.5	36.3	598.5
Liu PH 2014 [22]	R(2002-2012)	HR	Taiwan USA	108	62 ± 15	91/17	NA	57/51	NA	91/17/0	50/58	NA	0.9 ± 0.9 (mg/dL)	38 ± 5	19,532 ± 55,912 (ng/mL)
		TACE		108	61 ± 14	84/24	NA	56/52	NA	95/13/0	49/59	NA	1.0 ± 0.6 (mg/dL)	38 ± 4	32,725 ± 138,234 (ng/mL)
Liu K 2014 [17]	R(2003-2010)	HR	China	41	48.7 ± 10.2	NA	9.3 ± 2.7	41/0	12/21/3/0	35/6/0	32/9	NA	28.5 ± 11.0	39.2 ± 6.1	10/31
		TACE		72	49.7 ± 10.2	NA	10.5 ± 3.6	72/0	24/32/16/0	59/13/0	60/12	NA	29.3 ± 11.5	38.4 ± 5.1	16/56
Ye 2014 [24]	R(2007-2009)	HR	China	90	49.3 ± 10.7	81/9	6.9 ± 1.6	51/39	0/66/24/0	84/6/0	12/78	NA	NA	NA	48/42
		TACE		86	45.6 ± 10.2	80/6	6.5 ± 2.7	32/54	0/66/20/0	78/8/0	18/68	NA	NA	NA	38/48
Lee 2016 [13]	R(2000-2011)	HR	Korea	40	55.0 ± 12.9	30/10	20/20	NA	0/26/14/0	35/5/0	31/9	27/13	0.9 ± 0.9 (mg/dL)	40 ± 0.6	10,728 ± 25,073 (ng/mL)
		TACE		80	58.3 ± 10.5	67/13	10/70	NA	0/31/49/0	58/22/0	54/26	73/7	1.3 ± 1.3 (mg/dL)	36 ± 0.5	27,302 ± 71,902 (ng/mL)
Zheng 2016 [1]	R(2000-2008)	HR	China	96	51.9 ± 14.3	75/21	7.9 ± 2.2	2.4 ± 1.4	25/23/23/25	75/21/0	86/10	82/14	NA	NA	1120.6 ± 3930.7 (ng/mL)
		TACE		134	51.6 ± 13.3	98/36	8.0 ± 2.4	2.7 ± 1.8	31/32/33/38	101/33/0	117/17	118/16	NA	NA	1222.2 ± 2698.1 (ng/mL)
Wang 2016 [11]	R(2002-2014)	HR	HongKong China	745	305/440 (**≥**50/<50)	679/66	138/607	693/52	263/351/194/0	737/8/0	670/75	513/232	543/202 (**<**18.8/**≥**18.8)	47/698 (<35/**≥**35)	274/471
		TACE		604	319/285 (**≥**50/<50)	534/70	79/525	474/130	47/288/269/0	567/37/0	125/479	473/131	353/251 (**<**18.8/**≥**18.8)	130/474 (<35/**≥**35)	230/374

R (1996-1998): Retrospective study and the time of patients included in case-control cohort, *NA* Not applicable, *TACE* Transarterial chemoembolization, *HR* Hepatic resection

^a^The type of PVTT was performed by Cheng’s classification [26, 27]. ^b^ All means data from all included patients, of whom were in HR and TACE group partially

### Treatment regimens

HR and TACE were performed on patients in two groups. The description of the operative procedure for HCC with PVTT was the same in all included studies. En bloc resection, partial hepatectomy or hemi-hepatectomy could be performed in type I/II PVTT patients because the PVTT in these cases did not invade the edge of the resection range and was confined to the hepatic lobes or segments. If PVTT had extended to the main portal vein, considered type III PVTT, then hemi-hepatectomy combined with thrombectomy or main portal vein resection followed by reconstruction is recommended. For example, PVTT can be extracted out from the opened stump of the portal vein and the stump closed after flushing with blood flow and normal saline, confirming that no PVTT remains.

TACE was performed using Seldinger’s technique in all included patients. The number of TACE treatment cycles ranged from 1 to 7. The mean intermediate interval ranged from 4 to 8 weeks. The chemotherapeutic agents were varied among the included studies and included 5-fluorouracil (5-Fu), mitomycin (MMC), cisplatin, carboplatinum and epiadriamycin. Lipiodol and gelatin sponge (Gelfoam) was used as an embolic agent in all studies. None of the patients received other treatments, as shown in Table 2.

**Table 2:** Procedures of HR or TACE groups

Study		TACE		HR
	Duration and interval	Chemotherapeutic agents	Embolic agents	Methods and procedure
Fan 2001 [20]	Median 2 times (ranged 1-4)	5-fluorouracil (5-Fu) 1000 mg, mitomycin (MMC) 12 to20 mg, cisplatin or carborplatinum 80 mg	Lipiodol 20 ml	En-bloc resection, partial hepatectomy or hemihepatectomy Thrombectomy
Fan 2003 [19]	Median 2-3 times (ranged 1-7)	5-fluorouracil (5-Fu) 1000-1500 mg, cisplatin 80-100 mg, mitomycin (MMC) 8 to20 mg or doxorubicin 80 mg	Lipiodol 2-20 ml	En-bloc resection, partial hepatectomy or hemihepatectomy Thrombectomy
Cheng 2005 [18]	NA	Cisplatin, doxorubicin and mitomycin	Lipiodol	En-bloc resection, partial hepatectomy or hemihepatectomy Thrombectomy
Fan 2005 [21]	Ranged 1-7 times	5-fluorouracil (5-Fu) 1000-1500 mg, cisplatin 80-100 mg, mitomycin (MMC) 8-20 mg or epiadriamycin 40-60 mg	Lipiodol 5-20 ml	En-bloc resection, partial hepatectomy or hemihepatectomy Thrombectomy
Zhou 2011 [25]	Every 1-2 months for 2-5 cycles.	5-fluorouracil (500 mg /m^2^) and Adriamycin (30 mg/m^2^)	Gelatin sponge particles (1 mm^3^)	Hepatectomy plus thrombectomy
Peng 2012 [12]	Mean of 2.1 (ranged 1-5)	Carboplatin 300 mg, epirubicin 50 mg and mitomycin C 8 mg	Gelatin sponge particles (1 to 2 mm in diameter) Lipiodol 5 mL	Hepatectomy plus thrombectomy
Liu PH 2014 [22]	NA	Adriamycin 20–30 mg	Lipiodol 5-10 ml Gelfoam (2–3-mm strips)	En-bloc resection, partial hepatectomy or hemihepatectomy Thrombectomy
Liu K 2014 [17]	NA	Cisplatin 50-100 mg and epirubicin 20-40 mg	Lipiodol 5-20 ml	En-bloc resection, partial hepatectomy or hemihepatectomy Thrombectomy
Ye 2014 [24]	1 month intervals (ranged 1-7)	Doxorubicin 30-50 mg and cisplatinum 50-100 mg	Lipiodol 10-20 ml and gelfoam particles	En-bloc resection, partial hepatectomy or hemihepatectomy Thrombectomy
Lee 2016 [13]	Every 4 weeks	Doxorubicin 50 mg	Lipiodol	Hepatectomy plus thrombectomy
Zheng 2016 [1]	Mean of 2.9 (ranged 1–7)	NA	Gelatin sponge	Hepatectomy plus thrombectomy
Wang 2016 [11]	Intervals of 6 to 8 weeks	Doxorubicin hydrochloride 20-60 mg, and cisplatin 5 mg	Lipiodol 5-30 ml and gelfoam fragments	En-bloc resection, partial hepatectomy or hemihepatectomy Thrombectomy

### Overall survival

For all included 3129 HCC patients, the median OS ranged from 8 to 64 months in the HR group and from 5 to 32 months in the TACE group as reported in 10 studies [12, 13, 17–22, 24, 25] (Table 3). In the HR group, the 0.5-year OS rate varied from 45.9 to 46.8% but was reported in only 2 studies [19, 21]. The 1-year OS rate varied from 14.2 to 86.5%, the 2-year OS rate varied from 0 to 58.3%, the 3-year OS rate varied from 0 to 69%, and the 5-year OS rate varied from 0 to 69%. In the TACE group, the 0.5-year OS rate ranged from 34.2 to 34.6%, the 1-year OS rate ranged from 10.5 to 77.6%, the 2-year OS rate ranged from 0 to 17.4%, the 3-year OS rate ranged from 0 to 50%, and the 5-year OS rate ranged from 0 to 35%. Based on the preliminary data described above, the 0.5-, 1-, 2-, 3-, and 5-year OS rates were better for the patients receiving HR than those receiving TACE.

**Table 3:** Outcomes of therapy for HCC with PVTT

Study(year)	Treatments	Patients	Median OS(months)	6-month(%)	1-year(%)	2-year(%)	3-year(%)	5-year(%) of Survival rates
Fan 2001 [20]	HR	74	12	NA	53.9	NA	26.9	16.6
	TACE	18	5	NA	22.2	NA	5.6	0
Fan 2003 [19]	HR	19	10.3	45.9	14.2	0	0	NA
	TACE	41	7.1	34.2	12.2	0	0	NA
Cheng 2005 [18]	HR	7	8.0 (4.5-11.5)	NA	14.3	NA	NA	NA
	TACE	38	5.0 (4.4-5.6)	NA	10.5	NA	NA	NA
Fan 2005 [21]	HR	24	10.1	46.8	22.7	9.8	0	NA
	TACE	53	7.3	34.6	11.8	0	0	NA
Zhou 2011 [25]	HR	38	10	NA	47.0	NA	22.0	NA
	TACE	10	7	NA	20.0	NA	0	NA
Peng 2012 [12]	HR	201	20.0 ± 1.8	NA	42.0	NA	14.1	11.1
	TACE	402	13.1 ± 0.6	NA	37.8	NA	7.3	0.5
Liu PH 2014 [22]	HR	108	64	NA	84	NA	69	69
	TACE	108	32	NA	71	NA	50	35
Liu K 2014 [17]	HR	41	21.5	NA	70.1	40.8	16.7	NA
	TACE	72	13.8	NA	44.8	17.4	7.5	NA
Ye 2014 [24]	HR	90	8.2	NA	28	20	15	NA
	TACE	86	7.0	NA	17.5	0	0	NA
Lee 2016 [13]	HR	40	19.9	NA	64.7	58.3	49.9	NA
	TACE	80	6.6	NA	46.2	15.4	7.7	NA
Zheng 2016 [1]	HR	96	NA	NA	86.5	NA	60.4	33.3
	TACE	134	NA	NA	77.6	NA	47.8	20.9
Wang 2016 [11]	HR	745	NA	NA	49.1	29.1	18.3	9.5
	TACE	604	NA	NA	27.6	11.3	6.8	4.6

**Fig. 2:**
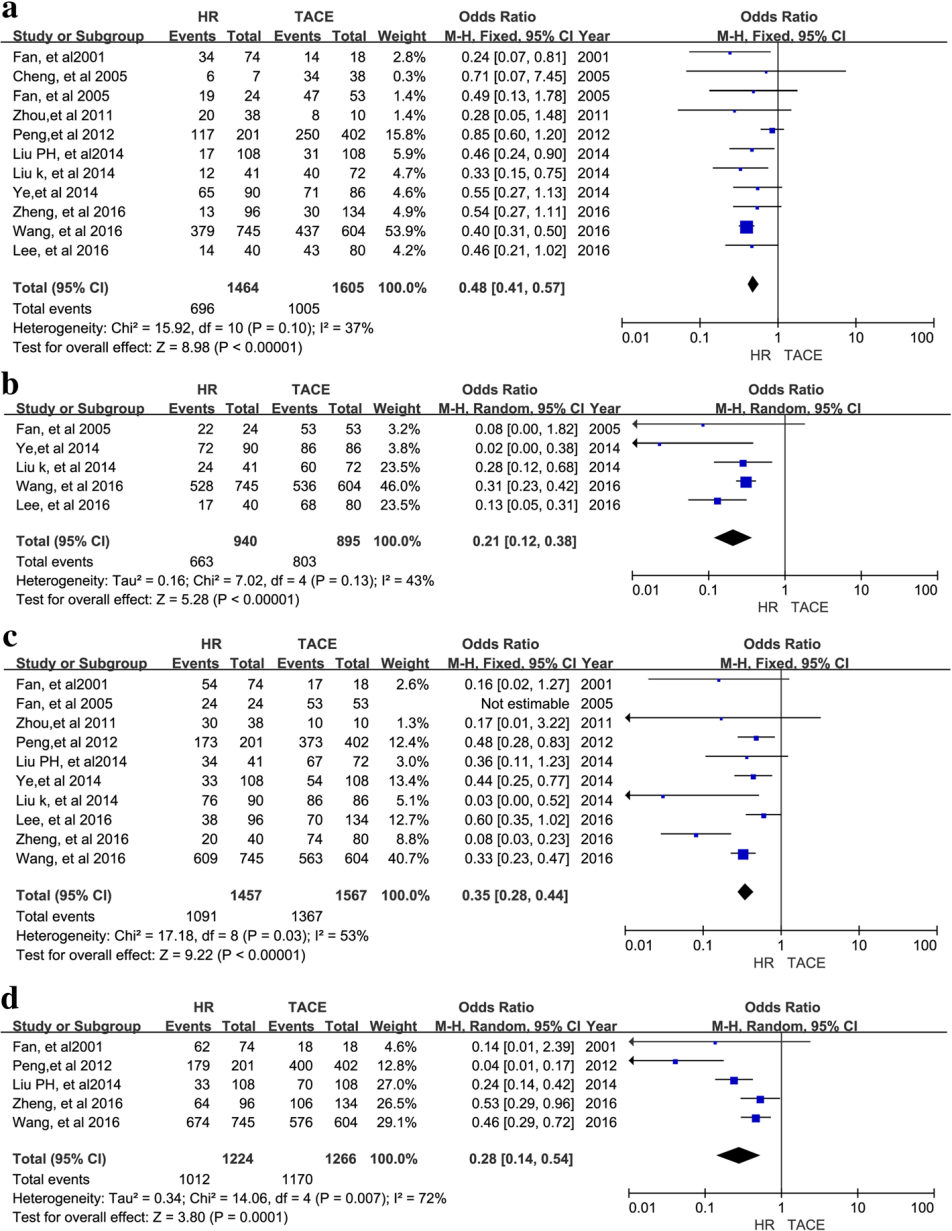
Forest plots for the comparison of ORs for OS in all included HCC patients with PVTT who received HR or TACE. Outcomes: **a** 1-year OS; **b** 2-year OS; **c** 3-year OS; **d** 5-year OS; A random effects model was used in the meta-analyses of the three outcomes

Eleven studies were included in the meta-analysis of 1-, 2-, 3-, and 5-year OS rates and the corresponding ORs. As shown in Fig. 2, the 1-year OS rates favored HR rather than TACE (OR = 0.48, 95% CI = 0.41–0.57, I2 = 37%, *P* < 0.00001; Fig. 2a) in all included studies, with 1464 patients undergoing HR and 1605 patients undergoing TACE. The 2-year OS rates (OR = 0.21, 95% CI = 0.12–0.38, I2 = 43%, P < 0.00001; Fig. 2b) were reported in 5 studies with 940 patients undergoing HR and 895 patients undergoing TACE. The 3-year OS rates (OR = 0.35, 95% CI = 0.28–0.44, I2 = 53%, P < 0.00001; Fig. 2c) were reported in 10 studies with 1457 patients undergoing HR and 1567 patients undergoing TACE. The 5-year OS rates (OR = 0.28, 95% CI = 0.14–0.54, I2 = 72%, *P* = 0.0001; Fig. 2d) were reported in 5 studies with 1224 patients undergoing HR groups and 1266 patients undergoing TACE. As shown in Fig. 2, the meta-analysis of RRs for OS indicated that the HCC patients with PVTT who underwent HR had significantly longer survival than those who underwent TACE.

### Subgroup analysis for outcomes of different types of PVTT

Our subgroup analysis uniformly used Cheng’s classification (Type I: tumor thrombus involving segmental branches of the portal vein or above; Type II: tumor thrombus involving the right/left portal vein; Type III: tumor thrombus involving the main portal vein trunk; Type IV: tumor thrombus involving the superior mesenteric vein) [26, 27]. As shown in Table 4, 7 of the 12 included studies indicated the OS for different types of PVTT in all patients [12, 13, 17, 20, 23–25]. Only one article reported OS rates for type IV PVTT at 1, 3, and 5 years in patients undergoing HR; these rates were 21.7%, 0%, and 0%, respectively, and were the same as those for patients undergoing TACE (1-year: 30.4%, 3-year: 4.3%, and 5-year: 0%, respectively; *P* = 0.371). Based on the data (Table 4) patients with type I and II PVTT in the first-order portal vein branch or lower-order portal vein branches had better results than patients with type III and IV PVTT in the main portal vein or the upper branches to the superior mesenteric vein. Therefore, Zheng et al. suggested that the OS of patients with type I PVTT was comparable to that of patients with type II PVTT (*P* > 0.05); similarly, the OS rates of patients with types III and IV PVTT were comparable (*P* > 0.05) [11].

**Table 4:** Outcomes of patients under HR or TACE for various PVTT types

Study(year)	Treatment	Type of PVTT	Patients	Median OS(months)	1-year(%)	2-year(%)	3-year(%)	5-year(%) of Survival rates
Fan 2001 [20]	HR	Type II	58	13.0	59.7	NA	27.4	8.8
		Type III	16	8.0	29.4	NA	14.3	11.1
	TACE	Type II	12	5.0	16.7	NA	8.3	0
		Type III	6	5.5	33.3	NA	0	0
Peng 2012 [12]	HR	Type I	27	NA	81.5	NA	51.2	37.9
		Type II	68	NA	46.3	NA	17.2	17.2
		Type III	83	NA	32.5	NA	3.6	3.6
		Type IV	23	NA	21.7	NA	0	0
	TACE	Type I	64	NA	41.1	NA	8.9	3.6
		Type II	136	NA	37.9	NA	6.0	0
		Type III	166	NA	36.1	NA	4.2	0
		Type IV	46	NA	30.4	NA	4.3	0
Liu K 2014 [17]	HR	Type I	12	30.0	100.0	66.7	30.0	NA
		Type II	21	18.2	66.7	32.6	0	NA
		Type III	8	8.9	25.0	0	0	NA
	TACE	Type I	24	19.7	74.8	30.8	12.8	NA
		Type II	32	10.8	25.9	9.7	0	NA
		Type III	16	7.4	25.0	0	0	NA
Lee 2016 [13]	HR	Type I	16	NA	71.4	54.5	54.4	NA
		Type II	8	NA	35.0	0	0	NA
	TACE	Type I	12	NA	50.0	25.0	16.7	NA
		Type II	14	NA	35.7	7.1	0	NA
Wang 2016 [11]	HR	Type I	122	14.7 (10.7-18.7)	57.2	36.1	21.0	10.0
		Type II	187	12.1 (10.0-14.2)	50.8	31.0	22.3	13.3
		Type III	171	6.2 (4.4-7.9)	36.3	17.4	8.2	2.6
	TACE	Type I	45	8.7 (4.1-13.3)	40.0	17.3	7.3	0
		Type II	187	5.3 (4.4-6.2)	25.1	9.5	5.3	4.5
		Type III	171	5.2 (3.7-6.6)	28.1	12.1	6.7	5.7

**Fig. 3:**
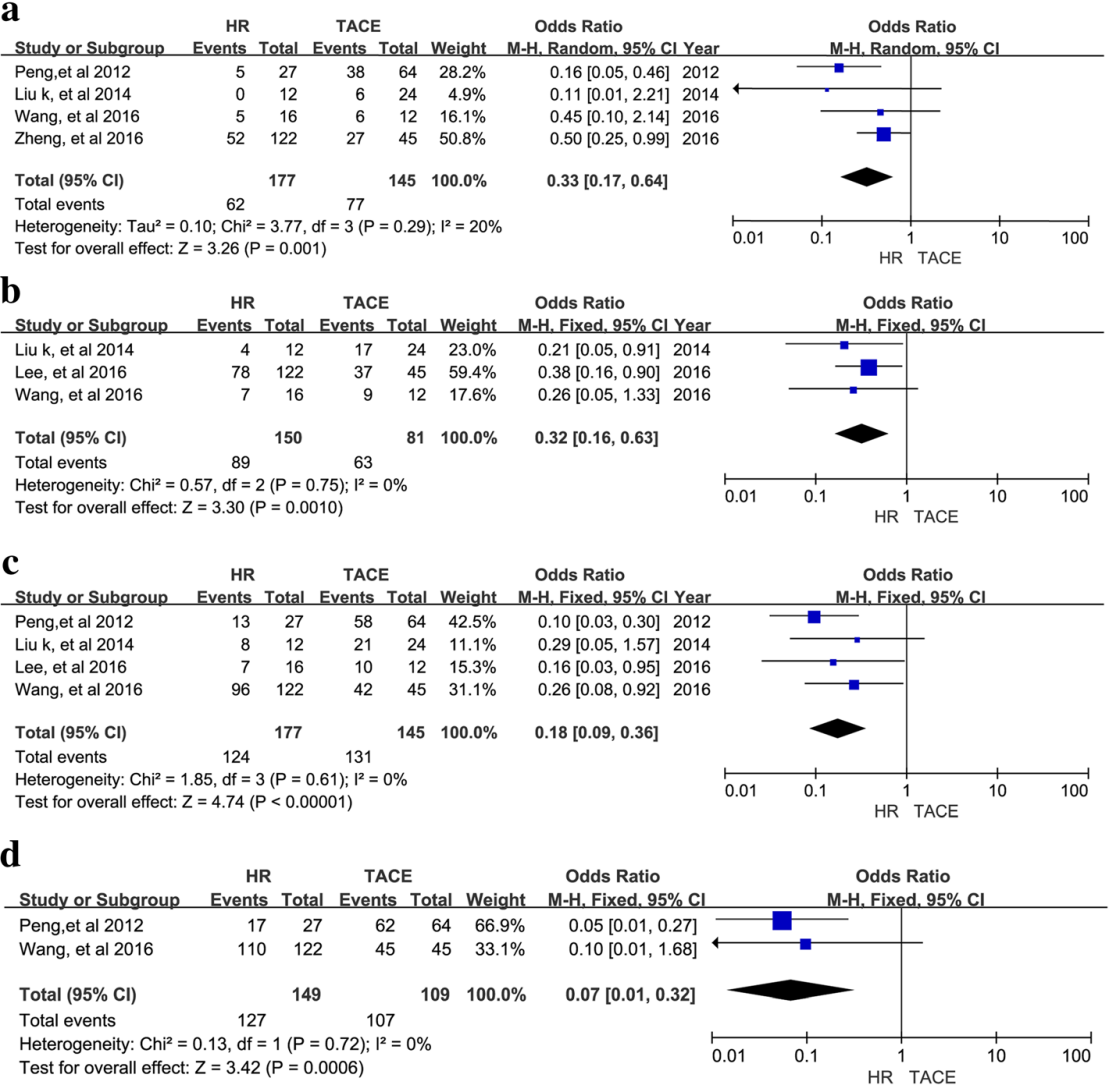
Forest plots for the comparison of ORs for OS in HCC patients with type I PVTT who received HR or TACE. Outcomes: **a** 1-year OS; **b**. 2-year OS; **c** 3-year OS; **d** 5-year OS; A random effects model was used in the meta-analyses of the three outcomes

**Fig. 4:**
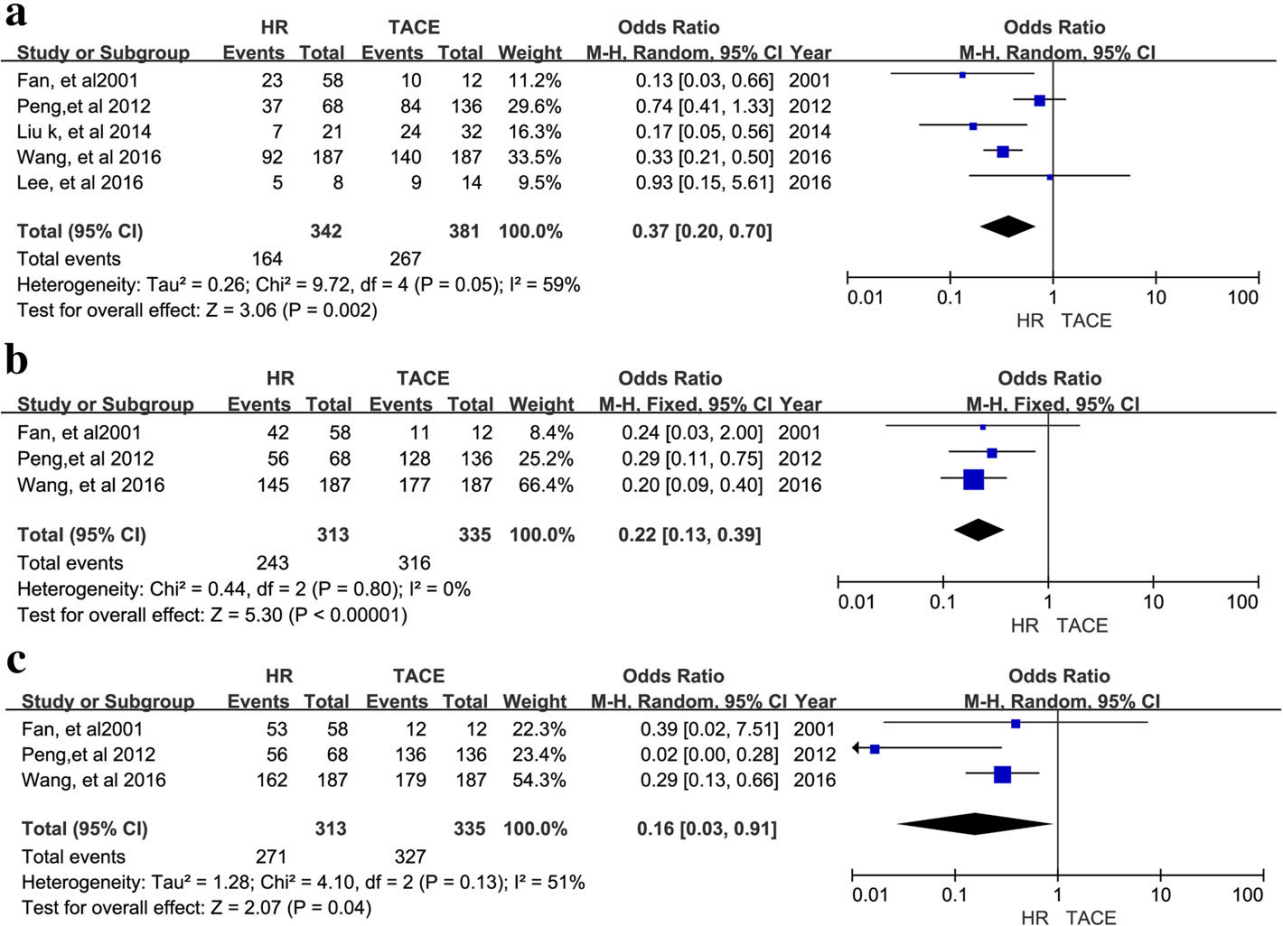
Forest plots for the comparison of ORs for OS in HCC patients with type II PVTT who received HR or TACE. Outcomes: **a** 1-year OS; **b** 3-year OS; **c** 5-year OS; A random effects model was used in the meta-analyses of the three outcomes

**Fig. 5:**
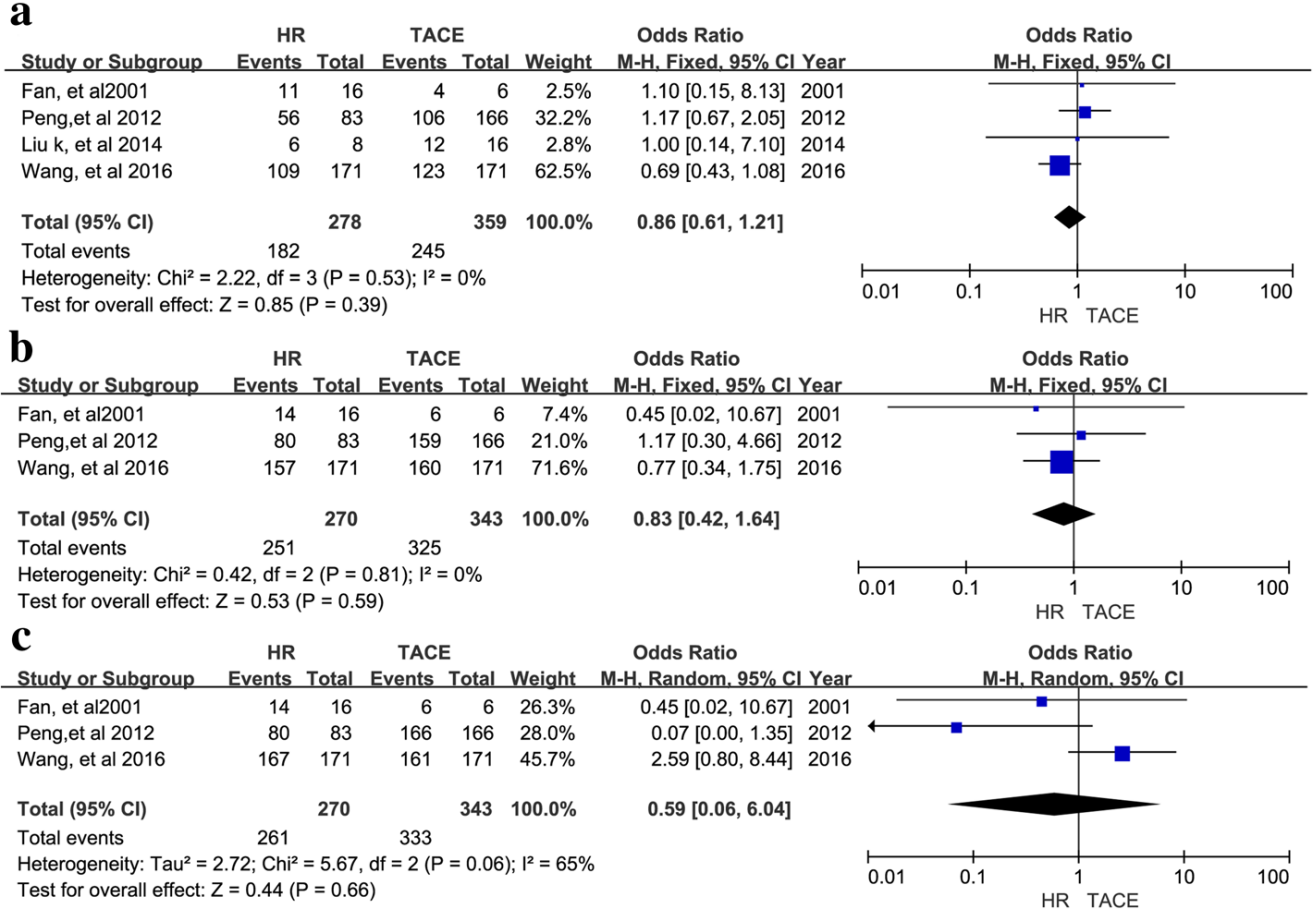
Forest plots for the comparison of ORs for OS in HCC patients with type III PVTT who received HR or TACE. Outcomes: **a** 1-year OS; **b** 3-year OS; **c** 5-year OS; A random effects model was used in the meta-analyses of the three outcomes

For type I PVTT, 4 studies reported 1-, 2-, 3-, and 5-year OS rates and corresponding ORs and were included in the meta-analysis. As shown in Fig. 3, the ORs for 1-, 2-, 3, 5-year OS for type I PVTT were better following HR than TACE (OR = 0.33, 95% CI = 0.17–0.64, I2 = 20%, *P* = 0.001, Fig. 3a; OR = 0.32, 95% CI = 0.16–0.63, I2 = 0%, *P* = 0.001, Fig. 3b; and OR = 0.18, 95% CI = 0.09–0.36, I2 = 0%, *P* < 0.00001, Fig. 3c; OR = 0.07, 95% CI = 0.01–0.32, I2 = 0%, *P* = 0.0006, Fig. 3d), respectively). For type II PVTT, 5 studies reported ORs for 1-, 3-, and 5-year OS and were included in the meta-analysis, (OR = 0.37, 95% CI = 0.20–0.70, I2 = 59%, *P* = 0.002, Fig. 4a; OR = 0.22, 95% CI = 0.13–0.39, I2 = 0%, *P* < 0.00001, Fig. 4b; OR = 0.16; 95% CI = 0.03–0.91; I2 = 51%, *P* = 0.04, Fig. 4c, respectively). Type I and II PVTT patients had a longer OS following HR than TACE. In contrast, the 1-, 3-, and 5-year OS for patients with type III PVTT were not significantly different following HR versus TACE. Correspondingly, the meta-analysis of ORs for 1-, 3-, and 5-year OS suggested that patients with type III PVTT can undergo either HR or TACE with similar results (OR = 0.86, 95% CI = 0.61–1.21, I2 = 0%, *P* = 0.39, Fig. 5a; OR = 0.83, 95% CI = 0.42–1.64, I2 = 0%, *P* = 0.59, Fig. 5b; OR = 0.59, 95% CI = 0.06–-6.04, I2 = 65%, *P* = 0.66, Fig. 5c, respectively).

#### Univariate and multivariate analyses of OS of PVTT patients

Whether potential correlations exist between OS and selected variables has not been reported. Thus, univariate and multivariate analyses of OS were performed for all patients in 7 studies [11–13, 21, 22, 24, 25]. In the univariate analysis, age, gender, BMI, race, cause of liver disease, preoperative antiviral therapy, portal hypertension, tumor size, tumor number, type of PVTT, Child-Pugh class, initial modalities of treatment, number of TACE cycles, AFP level ≥ 400 ng/mL, and NLR (neutrophil-lymphocyte ratio) ≥4 were found to predict poor OS across the 7 articles. Multivariate Cox proportional hazards regression analysis of all 7 studies indicated that type of PVTT and initial modalities of treatment may be significant prognostic factors for OS [12, 13].

## Discussion

There is a high incidence of PVTT in patients with advanced HCC, which is a significant prognostic factor for OS. Sorafenib is the only recommended treatment for PVTT according to BCLC C stage international guidelines for HCC patients. Recently, comprehensive treatments such HR and TACE [8, 28] have become available for HCC patients with PVTT, but use of these options remains controversial. This study is the first systematic review and meta-analysis to compare OS in HCC patients with PVTT receiving TACE or HR and provides a foundation for selecting appropriate clinical treatment. The analysis included 3129 HCC patients with PVTT. The results showed that HR was more effective and led to greater improvements in 1-, 2-, 3-, and 5-year OS for all included PVTT patients compared with TACE. Patients with type I and II PVTT experienced the greatest benefit.

Previous studies have suggested that HR is a safe and effective treatment for HCC with PVTT when patients are carefully selected. As reported in Ye et al. and Wang et al., PVTT patients undergoing HR have significantly higher OS than patients undergoing conservative treatment or TACE [23, 24]. Kokudo T et al. demonstrated that HR is associated with a longer OS than non-surgical treatment for patients with PVTT limited to the first-order branch [8]. The median survival time in the HR group was 1.77 years longer than that in the non-HR group (2.87 years vs 1.10 years; *P* < 0.001) and 0.88 years longer than that in the non-LR group (2.45 years vs 1.57 years; P < 0.001) in a propensity score-matched cohort. HR can eradicate both a main tumor and satellite tumors as well as PVTT to reduce the pressure on the portal vein, preventing the occurrence of intractable ascites and bleeding of esophageal varices, protecting liver function, and reducing tumor burden as well as intrahepatic and extrahepatic metastasis of HCC [29–31]. Thus, HR is considered to be potentially curative and is the preferred treatment for HCC patients with PVTT. However, several studies have reported that TACE is effective for the treatment of patients with advanced HCC with PVTT [7, 28, 32]. TACE is used to embolize arteries supplying blood and nutrients to tumors and has been used as adjunctive therapy for advanced HCC with PVTT, especially for preoperative and postoperative treatment [33, 34]. Previous reports have been inconclusive regarding whether HR or TACE has more benefits for PVTT. In this systematic review and meta-analysis, 1-, 2-, 3-, and 5-year OS showed greater improvements following HR than TACE for all included patients. These results further validated that some selected HCC patients with PVTT who have good liver function and no extrahepatic metastasis should be considered for HR. Notably, patients’ prognosis varied depending on PVTT type. Therefore, future studies should further clarify what types of PVTT respond better to HR or TACE.

The therapeutic effects of HR and TACE for various PVTT types were compared via subgroup analyses. For type I PVTT, defined as a tumor thrombus involving the segmental branches of the portal vein or above, the meta-analysis of 1-, 2-, 3, 5-year OS indicated that HR is more the appropriate treatment, producing a longer OS than TACE. Similarly, for patients with type II PVTT, the ORs corresponding to 1-, 3-, and 5-year OS suggested HR leads to better outcomes than TACE. These results agree with a study of a large cohort in Japan [8], wherein HR was associated with a longer OS than non-surgical treatment, including TACE, chemotherapy or transarterial chemoinfusion, ablation therapy, and best-supportive care, for patients with PVTT limited to the first-order branch of Vp1-3 [35], namely, those with type I or II PVTT. In the largest sample study conducted in China to date [23], the MST for type I and II patients (95% CI) undergoing HR was 15.9 (13.3–18.5) and 12.5 (10.7–14.3) months, respectively, while the corresponding figures for patients undergoing TACE were 9.3 (5.6–12.9) and 4.9 (4.1–5.7) months, respectively, which were significantly lower than those after HR (*P* < 0.05). This meta-analysis with high credibility than two respective studies illustrated that HR was the best treatment for type I and II PVTT patients with Child-Pugh A and selected B liver function. However, for type III PVTT, defined as a tumor thrombus involving the main portal vein trunk, this meta-analysis was unable to find differences in 1-, 3-, and 5-year OS between HR and TACE with high reliability. Based on these results, HR and TACE produce similar outcomes when treating patients with type III PVTT (*P* = 0.541). Although these patients can therefore choose either HR or TACE with good outcomes, most cannot receive HR because of PVTT extending to the main portal vein. Type III patients have poor liver function and high portal vein pressure; therefore, a single treatment is typically ineffective. TACE combined with radiotherapy should be given to type III PVTT patients according to Wang et al. [23] Preoperative adjuvant therapy such as TACE and radiotherapy could stage down type III PVTT to type I or II PVTT, which would then allow HR or TACE to be performed to achieve a longer OS [36]. Type IV PVTT, defined as a tumor thrombus involving the superior mesenteric vein, is rarely seen in HCC patients. Type IV PVTT is regarded as late-stage PVTT and corresponds to an extremely short OS. Although the use of HR for type IV PVTT remains controversial [37], Peng et al. [12] reported 1-, 3-, and 5-year OS rates of 21.7%, 0%, and 0%, respectively, for this treatment modality. However, no significant differences were found between HR and TACE for patients with type IV PVTT (*P* = 0.371). Thus, HR other than TACE should be performed for HCC patients with type I or II PVTT as opposed to those with type III or IV PVTT.

The study has several potential limitations. First, this meta-analysis contained numerous NRCT studies because there were no RCTs examining HR or TACE for the treatment of PVTT; therefore, selection bias was possible. Second there was significant heterogeneity between studies for some outcomes, which could have resulted from the quality of the NRCT studies, the small number of included studies especially in subset analyses, and the patient characteristics. The above limitations could have affected the results of this meta-analysis.

## Conclusions

In conclusion, the current systematic review and meta-analysis suggested that treatment of selected HCC patients with type I or II PVTT with HR may produce superior results to TACE. In contrast, there was no difference between HR and TACE for type III and IV PVTT. It is imperative to design additional rigorous multicenter RCTs with large samples to assess the use of HR and TACE in PVTT patients.

## Abbreviations

95% CIs: 95% Confidence intervals
BCLC: Barcelona clinic liver cancer
HCC: Hepatocellular carcinoma
HR: Hepatic resection
HRs: Hazard ratios
MST: Median survival time
NRCT: Nonrandomized Controlled Trials
ORs: Odds ratios
OS: Overall survival
PVTT: Portal vein tumor thrombus
RCT: Randomized controlled trials
TACE: Transarterial chemoembolization
